# Challenges and opportunities in combating viral hepatitis in India: insights from the Indian Hepatitis Summit 2025

**DOI:** 10.1186/s12919-025-00358-w

**Published:** 2026-01-29

**Authors:** Vivekanandan Shanmugam, John Ward, Sujatha Vijayakumar, Giten Khwairakpam, Cary James, Lokesh Shanmugam, Vaishnavi Girija, Bharat Bhushan Rewari

**Affiliations:** 1 Chennai Liver Foundation, Chennai, India; 2 Coalition for Global Hepatitis Elimination, The Task Force for Global Health, Decatur, GA USA; 3 Hashtag Medical Writing Solutions Pvt. Ltd., Chennai, India; 4 TREAT Asia, amFAR – The Foundation for AIDS Research, Bangkok, Thailand; 5 World Hepatitis Alliance, London, UK; 6 ICMR – National Institute of Epidemiology, Chennai, India; 7 Institute of Liver and Biliary Sciences, New Delhi, India

**Keywords:** Viral hepatitis, Hepatitis B virus, Hepatitis C virus, Liver disease, Public health, Vaccination, India

## Abstract

**Background:**

The world has committed to ending viral hepatitis as a public health threat as part of sustainable development goal 3.3. World Health Organization (WHO) has developed Global Health Sector Strategies (GHSS 2022–2030) to provide guidance to countries to end viral hepatitis by 2030. India instituted the national program for prevention and control of viral hepatitis (National Viral Hepatitis Control Program, NVHCP) in 2018 in alignment with the global perspective and strategic framework. The Chennai Liver Foundation conducted the first Indian Hepatitis Summit 2025 as a collective movement towards a hepatitis-free India.

**Methods:**

The summit was conducted on January 17–18, 2025, at Chennai. Healthcare professionals, policymakers, advocates, researchers, and civil society representatives gathered to share successes, discuss barriers, and address the urgent challenges of viral hepatitis and promote collaborative efforts to combat this silent epidemic in India.

**Results:**

The meeting was attended by 54 program faculty and 275 delegates from government agencies, national and international organizations, and public and private hospitals. The two-day agenda included 14 sessions, with 46 talks and four panel discussions. The summit gathered important lessons from global initiatives, surveyed the current landscape of viral hepatitis in India, shared the best practices from some parts of world including India, identified barriers to elimination initiatives, and discussed potential solutions to overcome these barriers. Discussions also included the developments in viral hepatitis screening, diagnosis, and treatment; the ways to tackle the stigma associated with the illness; and innovative measures to improve public health results. In collaboration with Indian Council of Medical Research (ICMR) and National Institute of Epidemiology (NIE), the Chennai Liver Foundation launched the Hepat App and a Hepatitis Online Course.

**Conclusion:**

The summit served as a platform to raise awareness about the importance of prevention, early diagnosis, and effective treatment options; and to delineate future directions for viral hepatitis elimination in India.

**Supplementary Information:**

The online version contains supplementary material available at 10.1186/s12919-025-00358-w.

## Introduction

India accounts for 11.6% of the global hepatitis B virus (HBV) and hepatitis C virus (HCV) burden, with about 29,800,000 people living with HBV and 5,500,000 people living with HCV [1]. The mortality rates of viral hepatitis and tuberculosis (TB) are strikingly identical, with the mortality being 1.3 to 1.4 million people globally [1]. However, the difference in viral hepatitis-related mortality is twofold: (a) it is increasing and (b) it is preventable due the availability of preventive vaccine for HBV and curative treatment for HCV [2].

The World Health Organization (WHO) ‘triple elimination initiative’ includes the elimination of mother-to-child transmission HBV, along with of human immunodeficiency virus (HIV) and syphilis [3]. The Global Health Sector Strategies (GHSS 2022–2030) aim to end HIV, HBV, HCV, and sexually transmitted infections (STIs) by 2030 [4]. Several countries are making remarkable progress towards the triple elimination goals. India has also instituted a national program for prevention and control of viral hepatitis (National Viral Hepatitis Control Program, NVHCP) in 2018 [5], despite ongoing efforts, the targets have not yet been met. With just five years to elimination goals, amplifying detection and treatment of viral hepatitis to a larger scale through innovative approaches and deliberate collaborations will help achieve the target in India.

Considering the global goal of viral hepatitis elimination by the year 2030, the window of opportunity to act and advance towards this goal is short and the need for actionable strategies for India is dire. At this juncture, Chennai Liver Foundation, a non-profit organization organized the first-ever Indian Hepatitis Summit attended by healthcare practitioners, researchers, policy makers, public health experts and industry leaders at Chennai, India. Indian Hepatitis Summit (IHS) provided a platform to collaborate and share knowledge, strategies, and best practices to combat viral hepatitis elimination in the country. IHS marks a new beginning to accelerate efforts to eliminate viral hepatitis through actionable strategies along with global and local partnerships.

### Strategic pathways to viral hepatitis elimination: lessons from global action plans

**Fig. 1:**
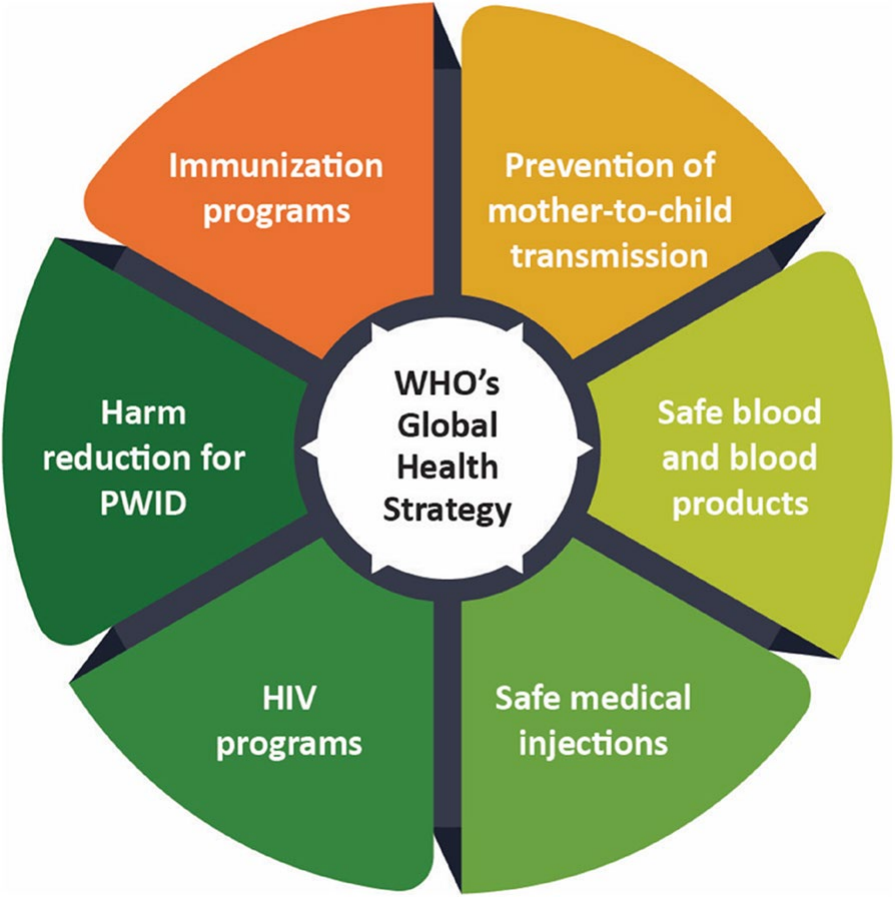
WHO’s Global Health Strategy to eliminate viral hepatitis, HIV, and STIs. HIV, human immunodeficiency virus; PWID, people who inject drugs; STIs, sexually transmitted infections; WHO, World Health Organization

Strategies from the global action, regional insights from the Southeast Asia Region (SEAR), and success stories from other low and middle income countries were discussed to understand the applicability of the elimination strategies in the Indian context. In SEAR, 61.4 million people are living with HBV infection and 9.1 million with HCV infection. Within the global burden, SEAR contributes 24% of viral hepatitis, 10% of HIV, and 16% of STIs of global burden. HBV and HCV infections account for two-thirds of the global burden of cirrhosis. In SEAR as well, more than half of the cirrhosis cases are due to HBV and HCV infections, with the HBV:HCV ratio of 0.9. WHO’s GHSS outlines the actions for eliminating HIV, HBV, HCV, and STIs (Fig. 1). These strategies are also being applied in India for viral hepatitis elimination.

Egypt has implemented effective HCV elimination programs via testing of all adults, awareness campaigns, electronic registries, and follow ups. The Local Hepatitis Elimination and Prevention (LHEAP) program enabled door-to-door HBV and HCV testing and referral to care in Pakistan.

**Fig. 2:**
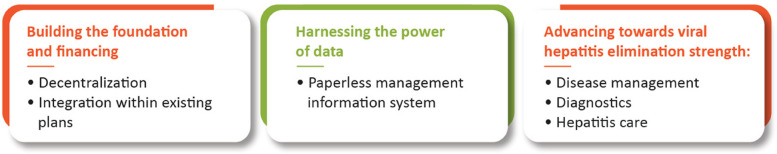
India’s comprehensive plan towards viral hepatitis elimination goals

India has made remarkable progress in its journey to viral hepatitis elimination (Fig. 2). Decentralization and integration with existing programs/schemes such as Maternal and Child Health Programs for pregnant women and babies, National AIDS (acquired immunodeficiency syndrome) Control program for high-risk groups, and blood bank for other populations has helped with financing and built a strong foundation for viral hepatitis elimination. The status neutral approach of HIV can be adopted to viral hepatitis for better prevention and treatment. NVHCP has adopted paperless management information system, designed for the Indian context, for efficient data-driven decision making. Key priorities and pathways to eliminate viral hepatitis by 2030 include strengthening disease management, diagnostics, and hepatitis care. Considering the gaps in testing and treatment uptake, accessibility challenges, and emerging evidence, there is also a need for regular updates in approach and management guidelines.

### Current landscape of viral hepatitis in India

**Fig. 3:**
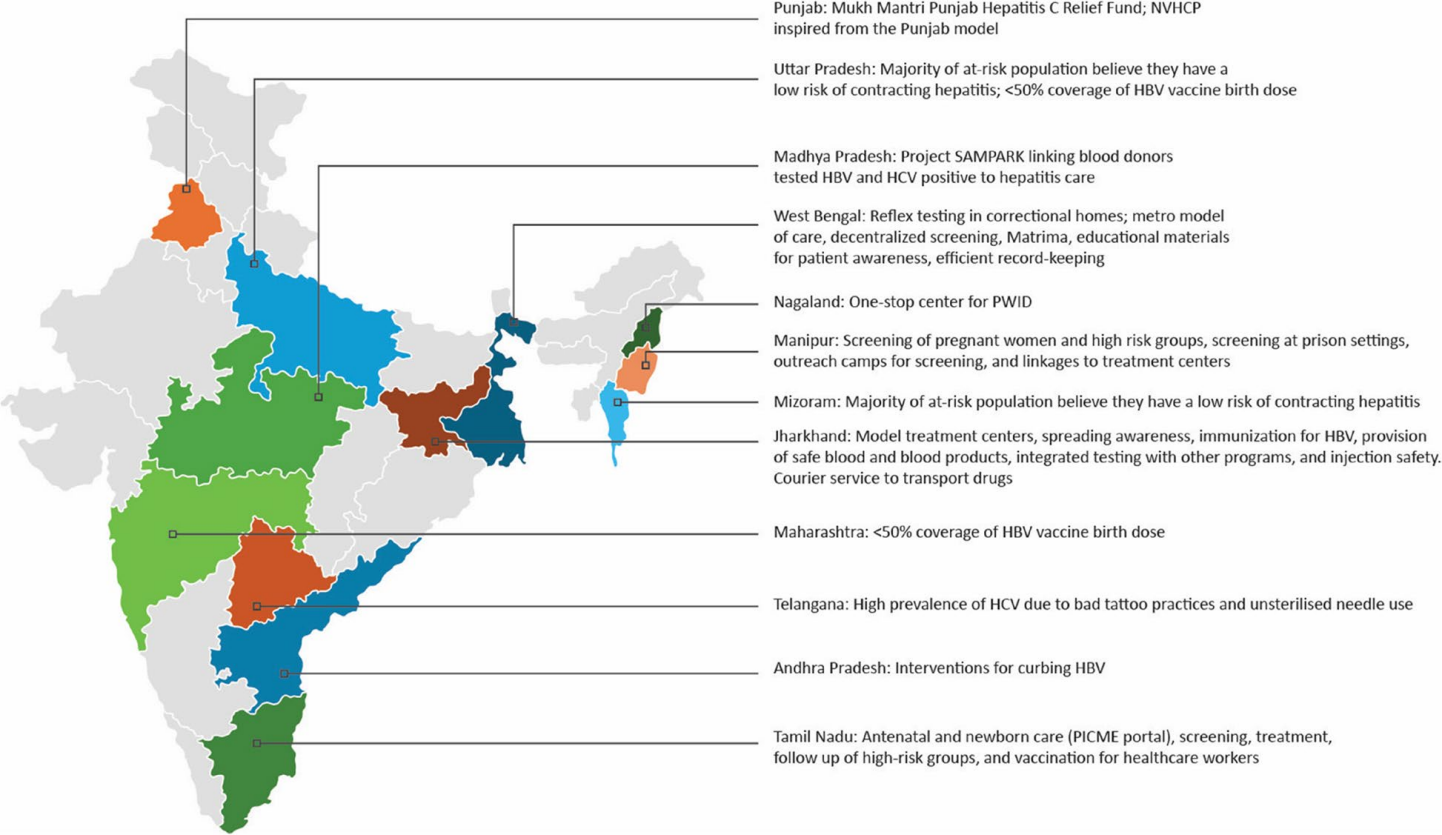
IHS discussions on the current status of viral hepatitis and state perspectives on enhancing linkages to care. HBV, hepatitis B virus; HCV, hepatitis C virus; NVHCP, National Viral Hepatitis Control Program; PICME, Pregnancy and Infant Cohort Monitoring and Evaluation; PWID, people who inject drugs

Several talks and a panel discussion reviewed the current status of viral hepatitis in India and the perspectives of different states on enhancing linkages to care (Fig. 3).

The NVHCP was launched in 2018 with clear goals and objectives. It is integrated into the National Health Mission and focuses on prevention, diagnosis, management, and capacity building. NVHCP was inspired by the program modelled in Punjab, which initially started to address the substantially high prevalence of viral hepatitis within this state, especially among intravenous drug users. The standard operating procedures of the Mukh Mantri Punjab Hepatitis C Relief Fund established an algorithm for diagnosis and treatment. Hepatitis C workshops provided training to about 120 primary care physicians from 25 centers across Punjab. As a part of the Punjab model, a system was designed to minimize patients lost to follow-up which alerted the doctor after two to three months of no contact, thus allowing the doctor to successfully follow up. Currently, NVHCP has 978 treatment sites across 711 districts throughout the country and has trained about 4900 master trainers at the national level, allowing approximately 0.3 million patients to have access to treatment. Collaborations with programs such as the National AIDS Control Program have allowed targeting control of HCV and treatment coverage across the country.

TREAT Asia has an important role in the elimination initiatives in India. TREAT Asia has models around same day HCV testing and treatment in people who inject drugs (PWID) which can be rolled out in collaboration. TREAT Asia can provide technical assistance for the elimination initiatives in India.

### Viral hepatitis in special populations

Individuals with HBV and HCV experience stigma and discrimination in every aspect of their life. The stigma is even more pronounced for individuals from certain communities.

#### Co-exiting mental health illness

Viral hepatitis often co-exists with depression. Patients with schizophrenia, borderline personality disorder, and other psychoses are less likely to be aware of HCV care and are more prone to stigma, thus delaying treatment. Awareness initiatives for mental health professionals are essential to educate them about screening at-risk patients and the impact of stigma.

#### Women health

Women health is an overlooked issue, with discrimination within the healthcare settings against women living with HIV, HBV, or HCV. Specific women-centric care plans for viral hepatitis do not exist. Screening and treatment centers are often inaccessible and situated away from the community, which should ideally be present in all parts of the country. Moreover, treatment of viral hepatitis in pregnant women may be challenging due to temporary reduction in alanine aminotransferase levels during pregnancy.

#### People with disabilities

People with disabilities and comorbidities such as intellectual disabilities, blood disorders, cancer, kidney failure, etc. face pronounced challenges in accessing health care. The VaccinEquity conducted for coronavirus disease 2019 (COVID-19) highlighted potential barriers to vaccination including app inaccessibility for persons with visual impairment; lack of accessible transport or ramps, binary queues, and washrooms; and mistreatment by staff at vaccination centers. All programs and interventions should be disability-inclusive to increase accessibility.

#### Individuals from the LGBTQIA + community and those who engage in sex work

Targeted community-led approaches and government-led initiatives include setting up camps in institutional settings, feasible timings according to work schedules of transgender and sex worker communities, and staff sensitization. Recommendations include gender-sensitive communication, destigmatization through awareness campaigns, peer support, and setting up helpline numbers.

#### People who inject drugs (PWID)

Mostly PWID do not seek professional hepatitis care, data on screening and treatment among these individuals is largely absent. For PWID, needle syringe programs, oral substitution therapy, reflex testing, and care coordinators can help mitigate risk of HBV and HCV infection. PWID should be educated about the possibility of re-infection after treatment, use of condoms for prevention, periodic re-testing to avoid diagnostic delays, and treatment for acute HCV re-infection.

### Barriers and facilitators to viral hepatitis elimination in India

During different sessions, several obstacles were identified while addressing the complexities of viral hepatitis diagnosis, care, vaccination, treatment, and elimination (Table 1). Major challenges include lack of awareness even among healthcare workers, poor recording and management of data, sub-optimal vaccine coverage due to multiple reasons, and patient loss to follow-up.

**Table 1:** Barriers to viral hepatitis elimination in India

Parameters	Barriers
Vaccination	• Low awareness among parents and nurses about HBV birth dose • Dearth of data to build a strategic plan • Sub-optimal coverage of birth dose of vaccine • Non-availability of vaccine
Diagnosis	• Asymptomatic nature of viral hepatitis • Lack of knowledge among the public and healthcare professionals about viral hepatitis prevention and services • Lack of infection status awareness among the majority of the people living with viral hepatitis • Lack of easily accessible testing and diagnostic confirmation • Stigma and discrimination • Point-of-care platforms are not fully utilized • High cost of tests in the private sector • Surveillance challenges to identify acute HCV infections
Treatment	• Good models of care for viral hepatitis not established • Lack of access to treatment centers • High costs and economic losses associated with travel to treatment centers
Follow-up	• Delay in evaluation process • Long treatment duration for HBV infection (life-long in most cases) • Challenges with maintenance of patient data
Others	• Limited availability of reliable epidemiological data for advocacy and programmatic actions • Limited funding due to competing health priorities (no international donors unlike HIV and TB) • Low community engagement • Inadequate political commitment • Lack of awareness among infected individuals and the general population • Lack of reach

HBV, hepatitis B virus; HCV, hepatitis C virus

While many potential facilitators to viral hepatitis elimination in India were discussed (Table 2), implementation requires political commitment by national and state level leadership. Discussions also revolved around the efficiency and performance of the steering committee of the NVHCP both at central and state level. These bodies design and allocate budget for the program. The government should be mobilized to be accountable for programs and community engagements in terms of funding and resource allocation. A shift in perspective within the political leadership and integration of private and public practices is essential for effective implementation of elimination plans.

**Table 2:** Facilitators of viral hepatitis elimination in India

Parameters	Facilitators
Vaccination	• Scale-up of universal access to hepatitis B birth dose vaccine and improved services for prevention of vertical transmission • Continuous investment in primary prevention • Pairing TB and polio vaccines with the birth dose of HBV • Increasing access to data from eVIN for the frontline workers and program managers for better tracking and coverage of the vaccination program • Adequate stock of vaccines in labor rooms • Training and education of nurses and parents to improve adherence to the HBV birth dose and completion of three doses • Tailoring awareness campaigns to cultural contexts • Leveraging experiential learning through gamification • Research to identify area-specific implementation gaps • Digital health records to identify missed children among migrants • Greater use of data to drive decisions for action • Mobile vaccination camps or “vaccine on wheels” • Introducing oral and aerosol HBV vaccines to increase uptake • Preventive hepatology clinics
Testing	• Access to quality-assured test/diagnostic products • Establishment of quality management systems at testing sites • Integrated training of healthcare workers • Reflex testing for HBV and HCV RNA on-site • Role of point-of-care HCV viral load in improving linkage • Dried blood spots specimens for viral load/serology • Low cost HCV core antigen rapid diagnostic test for confirmation of viral infection • Integrating HIV, TB, syphilis, HBV, and HCV screening (multi-disease platforms) to facilitate active testing • HCV self-testing to promote access to testing • Providing videos and learning material for the uptake of self-testing • Validating FDA-approved self-testing devices in the Indian population • Screening camps for convenience of testing • Setting up testing camps close to slum areas
Treatment	• Entecavir dose of 0.5 mg for optimal results • Development of curative drug regimens for hepatitis B virus • Non-exclusive voluntary licensing of drugs to allow generic manufacturing • Strategies to strengthen linkage of testing to care and streamline diagnostic and treatment services • National registry of healthcare practitioners • Increased access to viral hepatitis treatment
Others	• Greater public awareness of the importance of viral hepatitis B and C prevention, testing, and treatment • Strengthened community and civil society through awareness programs and systematic engagement • Training and mentorship of non-specialists and nurses to support decentralization • Simplified and decentralized service as well as integrated service delivery • Regular updates in management guidelines • Adequate political commitment • Implementation of plan by district level leadership • Government accountability for programs and community engagements in terms of funding and resource allocation • Sensitization of the private healthcare sector to the national practices and to share resources and data with funded centers • Integrating hepatitis care into the universal health coverage and primary health care frameworks • Door-to-door visits and personalized one-to-one interactions with community members • Patient support groups conducted in community halls, temples, mosques, or churches • Addressing patient lost to follow-up • Advance research agenda

eVIN, electronic vaccine intelligence network; FDA, Food and Drug Administration; HBV, hepatitis B virus; HCV, hepatitis C virus; HIV, human immunodeficiency virus; TB, tuberculosis

### Innovative solutions for viral hepatitis elimination

**Fig. 4:**
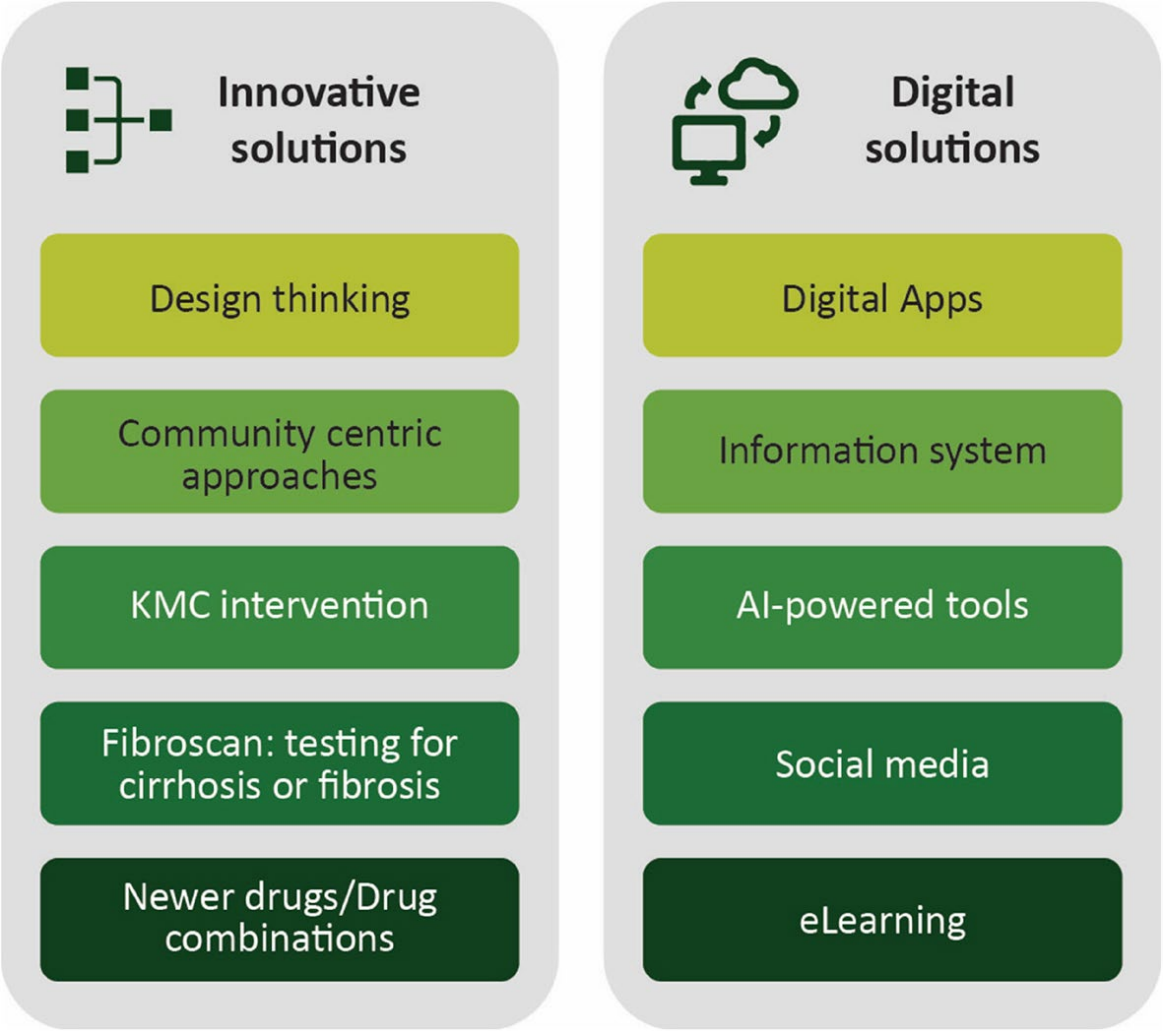
Solutions for viral hepatitis elimination in India. AI, artificial intelligence; KMC, kangaroo mother care

Several innovative solutions like design thinking in public health, integrated care models, and community-centric approaches were also discussed as potential solutions for overcoming obstacles (Fig. 4).

#### Design thinking

Design thinking revolves around four aspects—listening, observing, reading, and dialoging, which play a role in interacting with patients and other key actors who fall in the line of care pathway of viral hepatitis elimination. Framing and execution of public health strategies to achieve the intended goals could be explored. The example of community-based street plays was discussed as an impactful way for spreading awareness in rural areas.

#### Community-centric approaches

Community-centric approaches are often more effective than institutional-based service delivery for early detection and prevention, improving accessibility and affordability, and reducing stigma and discrimination. Community Network for Empowerment (CoNE) has initiated same day test-and-treat models and is currently running a vaccination campaign among PWID.

#### Industry perspectives

In the industry perspectives on innovations in hepatitis care, Transient elastography was discussed as a point-of-care innovation for early diagnosis since cirrhosis or fibrosis may not be apparent through screening tests for viral hepatitis. Likewise, the applicability of drugs against HCV infections such as glecaprevir/pibrentasvir in India was also discussed. In non-cirrhotic patients, glecaprevir/pibrentasvir has shown a 98% cure rate in HCV genotype 3 with only 8 weeks of treatment; 84% cure rates have been documented in certain cases with 4 weeks of treatment. However, uptake in India is low due to regulatory issues including patent restrictions and exclusion of India as a licensed territory for manufacturing glecaprevir/pibrentasvir. Patients may prefer traditional remedies rather than seeking modern medicine treatment for liver diseases in India.

#### Innovations from institutions

All India Institute of Medical Sciences (AIIMS) Rishikesh has adopted innovative approaches like establishing preventive hepatology clinics in 2021 and organizing fatty liver screening camps. Capacity Building efforts include Liver Induction Training and Update Program (LIT-UP) for healthcare practitioners. Project micro-elimination of HCV in district prison utilized a drone for transport of tests and treatments, and telemedicine sessions for the inmates. Hepatitis C treatment was delivered to 10 inmates using a drone at Uttarakhand’s Haridwar District Jail. Likewise, blood samples were collected from these inmates and sent to the hospital for testing. This initiative provides healthcare access to inmates without the need to transport them to hospitals. Medical professionals can also attend to inmates directly via telemedicine sessions.

### Digital solutions

Several talks focused on digital solutions for data accuracy, public health program implementation, awareness programs, and capacity building (Fig. 4).

#### Digital applications

Digital applications, currently under design, will reflect real-time data and next course of action for each beneficiary in the system. The aims of the app are to keep the language simple and easy to understand, and to increase acceptance and uptake at the beneficiary’s end.

The Hepat App by Chennai Liver Foundation, launched during the summit, can be used for field data collection and surveillance for diabetes mellitus, hypertension, and hepatometabolic diseases. It is a one stop solution to screen, diagnose, treat, and follow-up. The app is designed to operate at all levels—community, mobile clinics, and primary care hospitals. It can work offline. Moreover, it is FHIR (Fast Healthcare Interoperability Resource) compatible and ABDM (Ayushman Bharat Digital Mission) ready.

#### Information system

A good information system allows for better day-to-day decision making, better control and oversight, and reduces administrative burden. Digital health often fails due to a number of reasons such as lack of consideration of the end user, unsuitable technology for upscaling, budget issues, and lack of constructive discussion if the pilot system fails. A National registry of healthcare practitioners could boost implementation of hepatitis care.

Building responsive, user-friendly systems for program managers and service providers will help transform data into actionable items. The NVHCP management information system program is a paperless tool featuring detailed clinical modules, decision support systems aligned with the national guidelines, and provides role-based access for users.

#### Artificial intelligence, telemedicine, and social media

Tools that can assist in strengthening public health systems include voice-to-text models for collation and storage of data, AI-powered chatbots for virtual triage and symptom assessment, drone technology for medical supply delivery in remote areas, mobile applications to assist with campaigns, and wearable devices for real-time health monitoring and early intervention. Technology assists with telemedicine and remote patient monitoring for people residing at remote or rural areas, or for remotely tracking and preventing complications in chronic patients. User acceptance can be boosted through incentive programs and engaging with local communities. Social media such as YouTube, Instagram, and Facebook are viable tools to spread awareness.

#### Education initiatives

Digital solutions can also be leveraged for training and education of healthcare professionals. The traditional classroom model of learning fails to fulfill the capacity building needs of nurses and doctors all over the country. For healthcare professionals, e-Learning has proven to be highly effective for capacity building. In collaboration with Indian Council of Medical Research (ICMR) and National Institute of Epidemiology (NIE), Chennai Liver Foundation has launched a course for health professionals on viral hepatitis aiming to train 10,000 professionals. The Chennai Liver Foundation’s platform for online and hybrid courses on hepatitis, liver disease, and cardiometabolic diseases can be accessed at: https://learn.hepat.app.

### Hepatocellular carcinoma

One session focused on hepatocellular carcinoma (HCC), particularly its epidemiology, risk factors, prevention strategies, and palliative care for patients with liver cancer. Despite the relatively low documented rate, mortality due to HCC is higher in India when compared to the global burden. Even so, these data may not represent true incidence and mortality. The burden of HCC is estimated to rise in the next 20–30 years due to the existing burden of chronic HBV with advanced liver disease. About 80%–90% of patients are unable to have curative treatment at the time of diagnosis.

Risk factors of liver cancer include HBV and/or HCV infection, non-alcoholic fatty liver disease (NAFLD), alcohol use and smoking, a family history of liver cancer, and certain environmental toxins. Vaccination programs and treatment of HBV and HCV have reduced the incidence of HCC. HCC with a viral etiology can be prevented through the following strategies: full dose of HBV vaccinations, prevention of viral infection transmission from mother to child, blood and injection safety, harm reduction, and proactive viral hepatitis testing and treatment.

Merging preventive hepatology with alcohol use disorder clinics significantly helps in treating liver disease, reducing long term mortality, and achieving better outcomes. Tailors and barbers can potentially help identify risk factors such as a greater waist circumference and skin manifestations depicting insulin resistance. This can serve as an innovative screening strategy.

#### Palliative care for patients living with cancer

Palliative care starts right after diagnosis and is integrated into disease management via information on pain and symptom management, support related to the expected prognosis, and removal of barriers faced by patients and caregivers. Integrated palliative care improves the physical, psychological, social, and spiritual quality of life, and improves communication with the caregiver as well as the treating professional. India has a National palliative care helpline to connect patients and families with serious illnesses to the nearest center: 1800–202–7777.

### Future direction

Cohesive actions of the government, healthcare providers, partners, donors, and communities and collaborative multi-sector partnerships involving inclusive engagement with communities and affected individuals, supported by strong government leadership will help India accelerate towards achieving all-inclusive, equitable access to viral hepatitis prevention, diagnosis, treatment, and care services. The two-day discussions culminated in the following actionable strategies that the stakeholders can employ in India to accelerate the efforts towards viral elimination.

All stakeholders will work cohesively to:

- Support and strengthen the implementation of India’s national strategy throughout the country at a scale that ensures benefits from its provisions to the entire population
- Continue increasing coverage of timely birth dose of hepatitis B vaccine and other interventions to prevent the mother-to-child transmission of HBV to reduce the prevalence of hepatitis B in children less than five years old to ≤ 0.1%
- Engage with manufacturers to ensure a robust supply chain to avoid shortages in affordable hepatitis B vaccines, viral hepatitis diagnostics, and medicines
- Participate in operational and implementation research and contribute innovations to update and implement strategies and policies thus increasing access to viral hepatitis treatment that is anchored on evidence-based guidelines from WHO and international liver associations
- Ensure that communities and persons with lived experience play a meaningful role in the design and implementation of programs to ensure health equity, maximize efficiencies, combat stigma, and increase impact
- Promote knowledge dissemination for monitoring access to the data on viral hepatitis prevention, diagnosis, and treatment to reduce new infections and deaths caused by viral hepatitis.

Clinicians and their professional organizations will commit to:

- Strengthen non-specialist provider skills to decentralize hepatitis care
- Improve linkage to and retention in care of those diagnosed with HBV and/or HCV infections
- Promote person-centered integration of viral hepatitis testing, treatment, prevention, and care with programs involving primary care, cancer, HIV, STIs, TB, non-communicable diseases, maternal and child health, and other services
- Ensure clinical settings are tailored to the needs of communities and provide a service free from stigma and discrimination.

Communities and civil society organizations will commit to:

- Raise awareness of viral hepatitis and the social, health, and economic benefits of hepatitis elimination
- Assist persons living with viral hepatitis and their families to overcome stigma and access viral hepatitis screening and services
- Support viral hepatitis services through demand generation, peer support, and strengthening linkage to and retention in care
- Play their role in the design and delivery of decentralized and community-based programs to ensure equity of access.

Private sector and industry will commit to:

- Invest in innovation for affordable and accessible diagnostic tools and treatments for viral hepatitis
- Collaborate with governments and non-governmental organizations to address gaps in service delivery, particularly in underserved areas
- Support public health campaigns to increase awareness and reduce stigma around viral hepatitis
- Develop innovative health financing models that reduce out-of-pocket expenses for individuals accessing treatment.

## Conclusion

The Indian Hepatitis Summit 2025 initiated a dialogue between different stakeholders invested in the viral hepatitis elimination goals in India. It identified challenges that hinder the progress towards the goal and solutions that can enable implementation of initiatives and programs. It also surveyed the current initiatives ongoing in India and suggested future directions to accelerate the elimination efforts.

## Supplementary Information

Supplementary Material 1.

## Data Availability

No new data were created or analyzed during this study. Data sharing is not applicable to this article.
