# Supplementary material for: Challenges and opportunities in combating viral hepatitis in India: insights from the Indian Hepatitis Summit 2025
Source: BMC Proc. 2026 Jan 29;20(Suppl 4):4. doi: 10.1186/s12919-025-00358-w (PMC12853636; doi:10.1186/s12919-025-00358-w)
Supplement: Supplementary file 1 — Supplementary Material 1. [file 12919_2025_358_MOESM1_ESM.docx]

**Supplementary Table 1. Agenda**

| **Name** | **Affiliation** | **Topic** |
| --- | --- | --- |
| **Session 1: Strategic Pathways to Viral Hepatitis Elimination**  Chair: 1. Dr. John W. Ward (Director, Coalition for Global Hepatitis Elimination)  2. Dr. B. S. Ramakrishna (Director & Senior Consultant, Gastroenterology, SIMS Hospital) | | |
| Dr. Po-Lin Chan | Regional Advisor for Hepatitis, HIV, and STIs, WHO | Regional Insights: Eliminating Viral Hepatitis in Southeast Asia |
| Prof. Radha Krishnan Dhiman | Director, Sanjay Gandhi Postgraduate Institute of Medical Sciences, Lucknow | The National Viral Hepatitis Control Program: Scope and Impact |
| Ms. Oriel Fernandes | Director, Clinton Health Access Initiative (CHAI) | Scale up of Viral Hepatitis Services in India: Success Factors & Lessons from Global Action |
| **Session 2: Expert Insights: Shaping the Future of Hepatitis Care** | | |
| Mr. Arun Jain | Founder and CMD of Intellect Design Arena Ltd, Founder of Mission Samriddhi | Design Thinking in Public Health: Innovative Interventions for Hepatitis |
| **Session 3: Panel Discussion: Addressing the Complexities of Viral Hepatitis Elimination**  Chair: Mr. Cary James (Chief Executive, The World Hepatitis Alliance) | | |
| Dr. Priya Abraham | Director, National Institute of Virology and Professor of Clinical Virology, CMC | Overcoming Diagnostic Barriers in Viral Hepatitis |
| Dr. Amit Goel | Professor & Head, SGPGI | Therapeutic Strategies and Management Complexities in Hepatitis Care |
| Dr. Rakesh Aggarwal | Director, JIPMER | Navigating Management Challenges in Special Populations |
| Dr. Kavita S. Lole | Scientist ‘G’ ICMR-National Institute of Virology | Role of Genomic Epidemiology in Viral Hepatitis Control |
| Dr. Arindam Ray | Country Lead, New Vaccines and Immunization Systems, Bill and Melinda Gates Foundation | Overcoming Barriers to Vaccination for Viral Hepatitis |
| **Session 4: Panel Discussion: Interactive Session Combating Stigma and Discrimination in Viral Hepatitis**  Chair: Ms. Jessica Hicks (Director, The World Hepatitis Alliance) | | |
| Mr. Anil Parmar | Vice President, United Way Mumbai | Investing in Communities to Address Stigma and Discrimination |
| Dr. R. Thara | Vice Chairman, SCARF | Integrating Mental Health in HIV and Hepatitis Care Frameworks |
| Ms. Kousalya Periasamy | Founder, Positive Women Network | Empowering Women to Combat Hepatitis-Related Stigma |
| Sharin D’Souza | Assistant Research Coordinator, iHEAR, Sangath | Building Inclusive Healthcare: Experiences of Disabled and LGBTQIA+ Individuals |
| **Session 5: Opportunities for Integrated Care Models in Hepatitis Management**  Chair: 1. Dr. Amit Goel (Professor & Head, SGPGI)  2. Dr. Jyotsna Sistla (Lead - HIV & Hepatitis, WJCF) | | |
| Dr. B. B. Rewari | Visiting Professor (Epidemiology) & Public Health Specialist, ILBS | Navigating Hepatitis and HIV Co-Infection: Challenges and Innovations |
| Dr. Viswajeet Kumar | Deputy Director, Health and Family Welfare Department, Government of Tamil Nadu | Implementation Research: Paving the Way for Transformative Care Models |
| Dr. Reshu Agarwal | Technical Lead, HIV & Hepatitis , WHO India | WHO Guidelines: A Framework for Hepatitis Elimination |
| Dr. Kimberly Green | Global Director, Primary Health Care, PATH | Collaboration and Coalition-Building for Hepatitis Elimination |
| **Session 6: Lessons from the Field: Focusing on Real World Experiences in Hepatitis Care**  Chair: 1. Ms. Rachel Halford (President, World Hepatitis Alliance [WHA])  2. Dr. Malarvizhi (HOD, Medical Gastroenterology, Kilpauk Medical College) | | |
| Mr. Rajkumar Nalinikanta | President, Community Network for Empowerment (CoNE) | Community-Centric approaches to Hepatitis Elimination |
| Dr. K. S. Somasekhar Rao | Gastroenterologist and Hepatologist, Yashoda hospital | Our Journey: Experiences in Hepatitis Elimination |
| Dr. Saravanamurthy P. Sakthivel | Lead - Viral Hepatitis, PATH | Evaluating Acceptability and Usability of Hepatitis C Self-Testing |
| **Session 7: Industry Perspectives on Innovations in Hepatitis Care**  Chair: 1. Dr. B. Sumathy (Professor & Head , Department of medical gastroenterology, Stanley medical college and Hospital)  2. Dr. M. Balasubramanyam (ICMR Emeritus Scientist. Professor, Advisor & Senior Scientist, Madras Diabetes Research Foundation) | | |
| Dr. N. Murugan | Hepatologist, Apollo Hospitals | Point-of-Care Innovations: Fibroscan for early diagnosis |
| Mx. Yashwinder Singh | Public Health Expert | Glecaprevir/Pibrentasvir for Hepatitis C in India |
| **NOHep Medical Visionaries Forum**  Ms. Rachel Halford (President, World Hepatitis Alliance [WHA]) | | |
| Dr. Ajeet Singh Bhadoria | Associate Professor, AIIMS | Innovative Approaches through Preventive Hepatology at AIIMS Rishikesh |
| Dr. Shailesh |  | Technology and community work |
| Dr. Ganeshkumar Parasuraman | Scientist E, ICMR - NIE | Digital Health for effective Public Health Program Implementation |
| Dr. Jyotsna Sistla | Lead - HIV & Hepatitis, WJCF | Role of Information Systems |
| **Session 8: Panel Discussion: State Perspectives on Enhancing Linkages to Care**  Chair: 1. Dr. Asha Hegde (Director, South Asia, HIV - Hepatitis PATH)  2. Dr. Ajeet Singh Bhadoria (Associate Professor, AIIMS) | | |
| Dr. Pallav Bhattacharya | Advisor, State Task Force on TEVTHSH, West Bengal | West Bengal |
| Dr. Vinay Kumar | Joint Director (Immunization) & State Immunization Officer, Tamil Nadu | Tamil Nadu |
| Dr. Rajkumari Rosie | SNO of NHM, Manipur | Manipur |
| Dr. Praveen Kumar Karn | State Epidemiologist cum Assistant Nodal Officer, Jharkhand | Jharkhand |
| Ms. Lavanya A. | Research Associate III, SRM University | Hepatitis Evaluation to Amplify testing: Project Findings |
| **Session 9: Innovative Implementation Strategies for Viral Hepatitis Elimination**  Chair: 1. Ms. Rachel Halford (President, World Hepatitis Alliance [WHA])  2. Dr. Po-Lin Chan (Regional Advisor for Hepatitis, HIV, and STIs , WHO) | | |
| Dr. John W. Ward | Director, Coalition for Global Hepatitis Elimination | Success Stories From the Low- and Middle-Income Countries |
| Dr. Ramesh Reddy Allam | Medical Epidemiologist, CDC | The CDC’s Role in Advancing Hepatitis Elimination in India |
| Dr. N. Kumarasamy | Director, VHS Infectious Diseases Medical Centre | Clinical Trials in Addressing Challenges in Hepatitis Elimination |
| Ms. Mila Maistat | Senior Manager, Policy, Strategy and Market Access, Medicines Patent Pool | Global Access to Generic Hepatitis B and C Treatments: Opportunities for India |
| **Session 10: State Of The Art Lecture**  Chair: 1. Dr. B. B. Rewari (Visiting Professor (Epidemiology) & Public Health Specialist, ILBS)  2. Dr. C. E. Eapen (Professor and Head, Department of Hepatology, Christian Medical College) | | |
| Dr. Shiv Kumar Sarin | Director, Institute of Liver and Biliary Sciences | Navigating Hepatitis B Management: Treat Select or Treat All? |
| Dr. Shiv Kumar Sarin | Director, Institute Of Liver And Biliary Sciences | Policy and Advocacy for Reducing the Burden on Liver Cancer |
| **Session 11: Leveraging Communication Technology for Knowledge and Awareness**  Chair: 1. Mr. Cary James (Chief Executive, The World Hepatitis Alliance)  2. Dr. S. Lokesh (Scientist E, ICMR - NIE) | | |
| Dr. Prantar Chakrabarti | Consultant Hematologist, Zoho Corporation | Harnessing Technology to Strengthen Public Health Systems |
| Dr. Santhosh Jacob | Director, Orthopedics and sports injury management, Be Well Hospitals | Social Media as a Tool to Tackle Public Health Challenges |
| Dr. Ganeshkumar Parasuraman | Scientist E, ICMR - NIE | Professional Development through Learning Management Systems |
| **Session 12: Panel Discussion: India’s Path to Hepatitis Elimination: Challenges and Future Directions**  Panelists: 1. Dr. Rakesh Aggarwal (Director, JIPMER)  2. Dr. B. B. Rewari (Visiting Professor (Epidemiology) & Public Health Specialist, ILBS)  3. Dr. Po-Lin Chan (Regional Advisor for Hepatitis, HIV, and STIs , WHO)  4. Ms. Rachel Halford (President, World Hepatitis Alliance [WHA])  5. Dr. John W. Ward (Director, Coalition for Global Hepatitis Elimination)  6. Dr. Reshu Agarwal (Technical Lead, HIV & Hepatitis , WHO India)  7. Mr. Giten Khwairakpam (Program Manager, Community and Policy, TREAT Asia/amFAR) | | |
| **Session 13: A Generation Free From HBV: Eliminating Mother-To-Child Transmission in India**  Chair: 1. Dr. C. E. Eapen (Professor and Head, Department of Hepatology, Christian Medical College)  2. Yuhui Chan (Senior Manager, Strategic Planning and Operations, Global Hepatitis program, CHAI) | | |
| Dr. Vinay Kumar | Joint Director (Immunization) & State Immunization Officer, Tamil Nadu | Immunization as the Pillar of Viral Hepatitis Elimination |
| Dr. Asha Hegde | Director, South Asia, HIV - Hepatitis PATH | Achieving Triple Elimination: What Will it Take For India? |
| Dr. Tanvi Khera | Senior Program Lead, Product Development, Vaccine R&D, IAVI | Accelerating Hepatitis B Birth Dose Coverage: Challenges and Opportunities |
| Dr. Pallav Bhattacharya | Advisor, State Task Force on TEVTHSH, West Bengal | Driving Triple Elimination in West Bengal: Progress Milestones & Lessons Learnt |
| **Session 14: Hepatocellular Carcinoma**  Chair: 1. Dr. Premkumar (Assistant Professor, Medical Gastroenterology, Madras Medical College)  2. Dr. P. Piramanayagam (Consultant, Medical Gastroenterology, Apollo Hospital) | | |
| Dr. Murugan | Hepatologist, Apollo Hospitals | Hepatocellular Carcinoma: Epidemiology and Emerging Insights |
| Dr. Ajeet Singh Bhadoria | Associate Professor, AIIMS | Risk Factors and Prevention Strategies for Hepatocellular Carcinoma |
| Dr. Prakash Fernandes | Head, Palliative Care Partnerships, Cipla Foundation | Palliative Care: Addressing the Needs of Hepatitis and Liver Cancer Patients |

AIIMS, All India Institute of Medical Sciences; amfAR, The Foundation for AIDS Research; CDC, Centers for Disease Control and Prevention; CHAI, Clinton Health Access Initiative; CMC, Christian Medical College; CMD, Chairman & Managing Director; CoNE, Community Network for Empowerment; HBV, Hepatitis B virus; HIV, human immunodeficiency virus; HOD, Head of the Department; IAVI, International Acquired Immunodeficiency Syndrome Vaccine Initiative; ICMR, Indian Council of Medical Research; iHEAR; Initiative for Health Equity, Advocacy and Research; ILBS, Institute of Liver & Biliary Sciences; JIPMER, Jawaharlal Institute of Postgraduate Medical Education & Research; LGBTQIA+ lesbian, gay, bisexual, transgender, and queer, additional identities; NHM, National Health Mission; NIE, National Institute of Epidemiology; SCARF, Schizophrenia Research Foundation; SGPGI, Sanjay Gandhi Post Graduate Institute; SIMS, SRM Institutes for Medical Science; SNO, State Nodal Officer; SRM, Sri Ramaswamy Memorial; STI, sexually transmitted infections; TEVTHSH, Triple Elimination of Vertical Transmission of HIV, Syphilis & Hepatitis B; TREAT Asia, Therapeutics Research, Education, and AIDS Training in Asia; VHS, Voluntary Health Services; WHA, World Hepatitis Alliance; WHO, World Health Organization; WJCF, William J. Clinton Foundation
